# Effects of a Multicomponent Exercise Program in Physical Function and Muscle Mass in Sarcopenic/Pre-Sarcopenic Adults

**DOI:** 10.3390/jcm9051386

**Published:** 2020-05-08

**Authors:** Hyuma Makizako, Yuki Nakai, Kazutoshi Tomioka, Yoshiaki Taniguchi, Nana Sato, Ayumi Wada, Ryoji Kiyama, Kota Tsutsumimoto, Mitsuru Ohishi, Yuto Kiuchi, Takuro Kubozono, Toshihiro Takenaka

**Affiliations:** 1 Department of Physical Therapy, School of Health Sciences, Faculty of Medicine, Kagoshima University, Kagoshima 890-8544, Japan; nakai@health.nop.kagoshima-u.ac.jp (Y.N.); kiyama@health.nop.kagoshima-u.ac.jp (R.K.); 2 Graduate School of Health Sciences, Kagoshima University, Kagoshima 890-8544, Japan; reha_tommy@yahoo.co.jp (K.T.); p.taniguchi0601@gmail.com (Y.T.); na2.stch.or@gmail.com (N.S.); ayumi0924n.n@gmail.com (A.W.); yuto.kch55@gmail.com (Y.K.); 3 Tarumizu Municipal Medical Center Tarumizu Chuo Hospital, Kagoshima 891-2124, Japan; takenaka@tarumizumh.jp; 4 Department of Physical Therapy, Kagoshima Medical Professional College, Kagoshima 891-0133, Japan; 5 Center for Gerontology and Social Science, National Center for Geriatrics and Gerontology, Obu 474-5811, Japan; k-tsutsu@ncgg.go.jp; 6 Department of Cardiovascular Medicine and Hypertension, Graduate School of Medical and Dental Sciences, Kagoshima University, Kagoshima 890-8520, Japan; ohishi@m2.kufm.kagoshima-u.ac.jp (M.O.); kubozono@m.kufm.kagoshima-u.ac.jp (T.K.)

**Keywords:** muscle strength, sarcopenia, resistance training, randomized controlled trial

## Abstract

This study aimed to assess the effects of a multicomponent exercise program on physical function and muscle mass in older adults with sarcopenia or pre-sarcopenia. Moreover, we aim to standardize the exercise program for easy incorporation in the daily life of community-dwelling older adults as a secondary outcome. A single-blind randomized controlled trial was conducted with individuals (≥60 years) who had sarcopenia or pre-sarcopenia (*n* = 72). Participants were randomly assigned to the exercise and control groups. The exercise program consisted of 12 weekly 60-min sessions that included resistance, balance, flexibility, and aerobic training. Outcome measures were physical function and muscle mass. Assessments were conducted before and immediately after the intervention. Among the 72 participants (mean age: 75.0 ± 6.9 years; 70.8% women), 67 (93.1%) completed the trial. Group-by-time interactions on the chair stand (*p* = 0.02) and timed “up and go” (*p* = 0.01) tests increased significantly in the exercise group. Although the exercise group showed a tendency to prevent loss of muscle mass, no significant interaction effects were observed for cross-sectional muscle area and muscle volume. The 12-week exercise program improved physical function in the intervention group. Although it is unclear whether the program is effective in increasing muscle mass, a multicomponent exercise program would be an effective treatment for physical function among older adults with sarcopenia.

## 1. Introduction

Sarcopenia is defined as a general loss of skeletal muscle mass and strength and is considered a major health problem for older individuals [1,2]. In 2016, sarcopenia was recognized as an independent condition by the International Classification of Disease, Tenth Revision, Clinical Modification (ICD-10-CM), code (i.e., M 62.84) [3].

Over the last decade, several clinical diagnostic criteria for sarcopenia have been reported worldwide [4,5,6,7,8,9]. In 2010, the European Working Group on Sarcopenia in Older People (EWGSOP) published its recommendations for a clinical definition and consensual diagnosis criteria [4]. Subsequently, many cohort studies identified sarcopenia based on these criteria, which include a combination of muscle mass and strength, and physical function loss [10,11]. In Asia, the most widely utilized criteria for determining sarcopenia are based on the Asia Working Group for Sarcopenia (AWGS) consensus, published in 2014 [8]. 

According to a previous systematic review that utilized the EWGSOP definition, the prevalence of sarcopenia is 1%–29% in community-dwelling populations and 14%–33% in long-term care populations with regional and age-related variations [12]. In the Asian population (Taiwan), the prevalence of sarcopenia varied from 3.9%–7.3% [13]. According to the AWGS criteria, the prevalence is estimated to range between 4.1% and 11.5% in the general older population [14]. A previous review and meta-analysis showed that the pooled prevalence of sarcopenia based on AWGS criteria among Japanese community-dwelling older individuals is 9.9%; similar prevalence rates were found in older men (9.8%) and women (10.1%) [15]. The numbers of people who had sarcopenia increased to 11%–50% for those aged 80 or above [16]. Community-dwelling older adults with sarcopenia have the worse physical performance [13,17] and are associated with premature mortality [18]. Since almost 10% or more of older individuals may meet the criteria for sarcopenia, effective prevention and improvement strategies are necessary.

Much interest has focused on community-based interventions for treating sarcopenia. A current systematic review and meta-analysis showed positive effects of exercise and nutritional interventions for older individuals [19]. However, there is little evidence of these effects, and the literature concludes that the evidence quality ranges from very low to low [19]. Therefore, well-designed randomized controlled trials (RCTs) to assess the effects of exercise on physical function and body composition, especially muscle mass, should be promoted.

Few well-designed intervention studies with a sufficient sample size have been conducted on the effects of exercise programs on sarcopenia. There is no effective treatment for sarcopenia, but physical exercise seems to be highly effective at counteracting the decline in physical function, muscle mass, and strength associated with ageing. The primary outcome of the present RCT was to investigate the effects of a multicomponent exercise program on physical performance and muscle mass in community-dwelling older adults with sarcopenia or pre-sarcopenia. Furthermore, as a secondary outcome, we aimed to standardize this approach for community-dwelling older adults, which can be easily incorporated into their daily lives.

## 2. Methods

### 2.1. Study Design

This community-based intervention study was a single-blind randomized controlled trial (UMIN 000036614). The intervention programs were implemented between June and September 2019. All participants provided written informed consent; after baseline measurements, they were randomly allocated to a 12-week multicomponent exercise program group or a wait-list control group. The study was approved by the Ethics Committee of the Faculty of Medicine, Kagoshima University (#180273).

### 2.2. Participants and Selection Criteria

**Figure 1:**
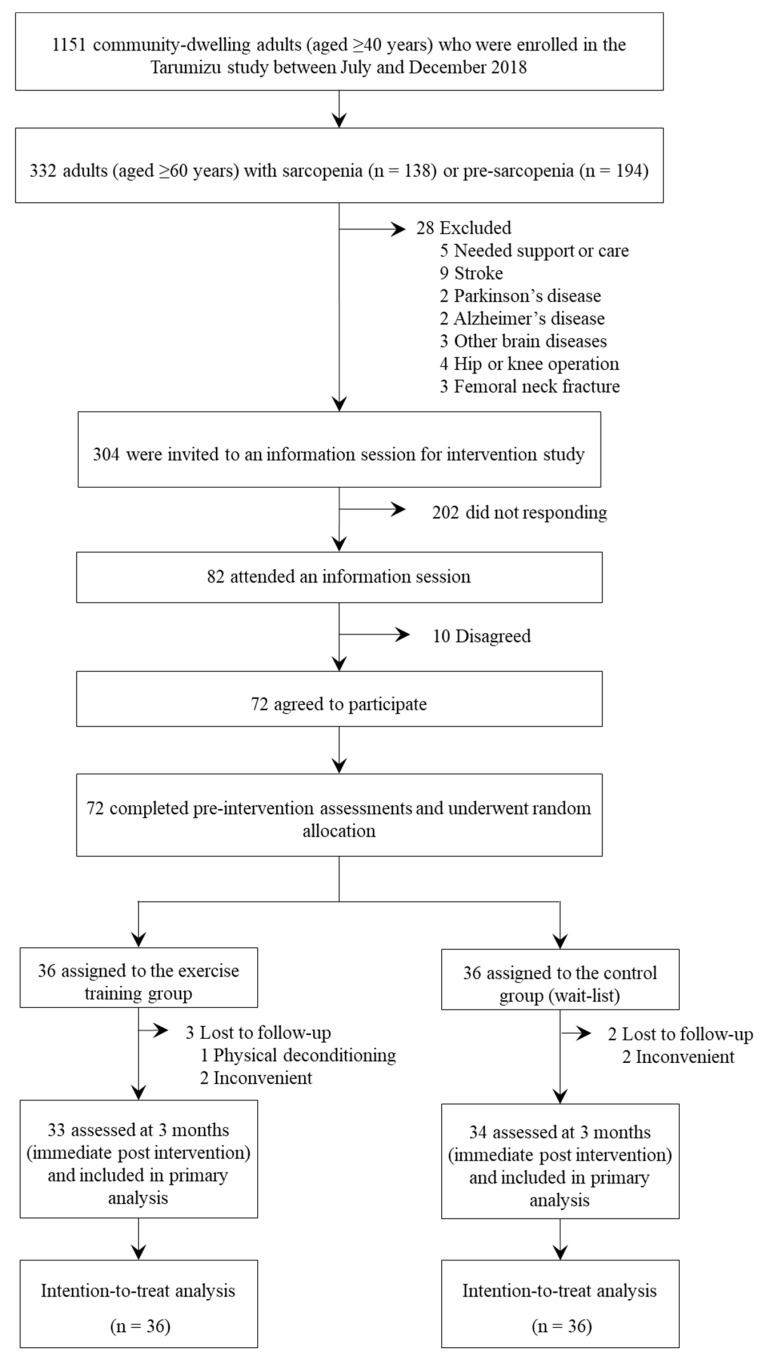
Flow diagram indicating participant progress through the trial.

We assessed 1151 community-dwelling adults aged 40 years or older who were enrolled in the Tarumizu study in 2018. Each participant was recruited from Tarumizu, a provincial city in Kagoshima Prefecture, Japan, between July and December 2018. Figure 1 presents the study flow. A total of 332 potential participants (≥60 years) with muscle mass loss (e.g., sarcopenia or pre-sarcopenia) were identified. Skeletal muscle mass loss was assessed by multi-frequency bioelectrical impedance analysis using the InBody 470 (InBody Japan, Tokyo, Japan). Appendicular skeletal muscle mass (ASM) was derived as the sum of the muscle mass of the four limbs, and the ASM index (ASMI, kg/m^2^) was calculated. Skeletal muscle mass loss was determined based on the AWGS criteria for sarcopenia: ASMI < 7.0 kg/m^2^ for men and <5.7 kg/m^2^ for women [8]. Participants with skeletal muscle mass loss and low physical function (low grip strength < 26 kg for men or <18 kg for women, and slowness, indicated by normal walking speed < 0.8 m/sec), were determined to have sarcopenia, and those with skeletal muscle mass loss without low physical function were determined as having pre-sarcopenia [20]. Individuals who did not use Japanese long-term care insurance and had a history of hip or knee operations, femoral neck fracture, stroke, Parkinson’s disease, Alzheimer’s disease, or other severe brain diseases, were excluded from the sample (Figure 1).

### 2.3. Intervention

Following randomization, individuals in the exercise training group participated in a progressive multicomponent exercise program over 12 weeks of supervised 60-min sessions that commenced in June 2019. The intervention consisted of resistance training, balance, flexibility, and aerobic exercises.

Thirty-six participants in the exercise groups were divided into two classes conducted by physiotherapists and instructors at a community center. Before each session, the participants checked their vital signs, including blood pressure, pulse rate, and self-reported physical condition. If vital signs were unsuitable, such as systolic blood pressure ≥ 180 mmHg, diastolic blood pressure ≥ 110 mm Hg, or resting pulse rate ≥ 110 bpm or ≤ 50 bpm, participants were asked to avoid exercise that day. Each session began with a brief warm-up involving stretching, followed by 25 to 30 min of resistance training, 20 to 25 min of balance and aerobic exercises, and 5 min of cool-down. Resistance training used a progressive sequence based on individual strength performance, starting with no resistance load (own weight) for the first two weeks. Progressive resistance was provided by resistance bands (TRIPLE TREE, Carbro Flavor USA Inc., CA, USA) that had five resistance levels. Individuals’ strength performance was tested every two weeks to determine the resistance load (intensity of resistance bands) and accordingly increase it for the next two weeks. In the strength performance test, participants determined their suitable resistance load at 12 to 14 on the Borg rate of perceived exertion scale [21], through ten repetitions of knee extensions. For each resistance exercise, participants completed up to ten repetitions of each movement, which included: (1) knee extension (quadriceps), (2) hip flexion (knee raises) (psoas major and iliacus), (3) hip internal rotation (gluteus medius and minimus), (4) elbow flexion and shoulder abduction (trapezius and rhomboid), (5) elbow flexion and trunk rotation (pectoralis major and oblique abdominis), (6) hip extension (gluteus maximus), (7) knee flexion (hamstrings), (8) hip abduction (gluteus medius), and (9) squat (quadriceps, gluteus maximus, and hamstrings). Balance training included a tandem stand, heel-up stand, one-leg stand, weight shifts, and stepping (anterior-posterior and lateral), to improve static and dynamic balance ability. Aerobic exercise consisted of anterior-posterior or lateral stepping repetitions for six minutes. The participants also performed daily home-based exercises, which were self-monitored using booklets, and were encouraged to record an exercise calendar. Exercise class attendance rate was calculated through the 12 exercise sessions as an exercise program adherence.

Participants in the wait-list control group (henceforth referred to as the control group) were asked to maintain their daily activities and attend a 60-min education class once during the trial period. The topic of this class was an irrelevant theme (e.g., preventing billing fraud).

### 2.4. Outcome Measures

#### 2.4.1. Physical Function

Grip strength and gait speed, performance on the chair stand test and timed “up & go” (TUG) were assessed to determine physical function and sarcopenia status, as recommended by EWGSOP2 and AWGS2 [22,23]. All assessments were administered by well-trained, licensed physical therapists.

Grip strength was measured in kilograms for the participant’s dominant hand, using a Smedley-type handheld dynamometer (GRIP-D; Takei Ltd., Niigata, Japan) [24].

Gait speed was measured in seconds using infrared timing gates (YW; Yagami Ltd., Nagoya, Japan). Participants were asked to walk on a flat, straight, 10 m-long walk path, at both usual and maximum gait speeds. Infrared timing gates were positioned at the 2 m mark and at the end of the path.

The chair stand test involved standing up from a sitting position and sitting down five times as quickly as possible without pushing off [25]. Physical therapists recorded the time a participant took to perform this action with their arms folded across their chests. The fifth repetition was recorded in seconds using a stopwatch (timed to 0.1 s).

In the TUG test, the participant rose from a standard chair, walked a distance of 3 m at a normal and safe pace, turned around, walked back, and sat down again [26]. Time was measured once in seconds, using a stopwatch.

#### 2.4.2. Cross-Sectional Muscle Area/Muscle Volume

**Figure 2:**
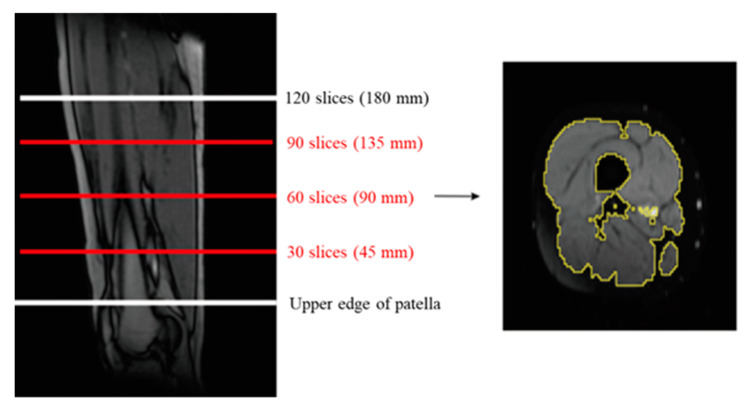
Cross-sectional muscle area of the thigh for segmentation and a sample segmented image.

Cross-sectional muscle area and muscle volume measurements were performed using magnetic resonance imaging (MRI), which was performed using a 1.5T MRI MAGNETOM Essenza (Siemens Healthcare, Germany). Imaging of both thighs was performed before and after the intervention period in a supine position with both legs extended. A total of 120 consecutive T1-weighted axial slices with 1.5 mm slice thickness were acquired from the upper edge of the patella. Three levels of cross-sectional muscle area (m^2^), lower, middle, and upper, were calculated from the right thigh. The lower level was calculated using 30 slices from the upper edge of the patella (45 mm proximal along the thigh). The middle and upper levels were determined using 60 (90 mm proximal along the thigh) and 90 slices (135 mm proximal along the thigh) from the upper edge of the patella, respectively. Muscle volume of the right thigh (cm^3^) was calculated using 60 consecutive slices between the lower and upper level slices (Figure 2). Image-J (NIH, USA, version 1.3) software was used to analyze the MRI images.

### 2.5. Statistical Analysis

The sample size was calculated using G^⋆^Power software (version 3.1.9.2) based on a previous study [27], which demonstrated that at least 28 participants were needed for each group to detect a 15% increase in physical functioning. We included 20% more patients in each group because of dropouts observed in our previous studies. The alpha error was defined as 0.05, with a power of 80%. Data have been presented as mean ± standard deviation (SD). All outcome data including physical function and muscle mass were assessed as normally distribution using the Kolmogorov–Smirnov test. Analysis of the intervention effects on outcomes was conducted according to the intention-to-treat principle, with the expectation-maximization algorithm estimation to substitute missing data. Outcome changes were verified by the Student’s *t*-test for paired data in each group. The repeated-measures analysis of variance (ANOVA), with group-by-time interaction, was used to evaluate the intervention effects. Data entry and analysis were performed using IBM SPSS Statistics for Windows (version 25.0). A *p*-value of <0.05 was considered statistically significant.

## 3. Results

### 3.1. Participant Characteristics at Baseline

Figure 1 summarizes the study flow. We screened 72 participants who were eligible and randomized. Participant characteristics and comparisons of baseline assessments between participants in the exercise and the control groups have been presented in Table 1. There were no significant differences in any of the characteristics and outcome measures between the exercise and control groups.

### 3.2. Exercise Program Adherence and Adverse Events

Among the 72 randomized participants, 67 (93.1%) completed the trial. The mean participation rate was 81% for the 12 exercise sessions. No adverse events related to the intervention were reported.

### 3.3. Sarcopenia-Related Physical Function

**Figure 3:**
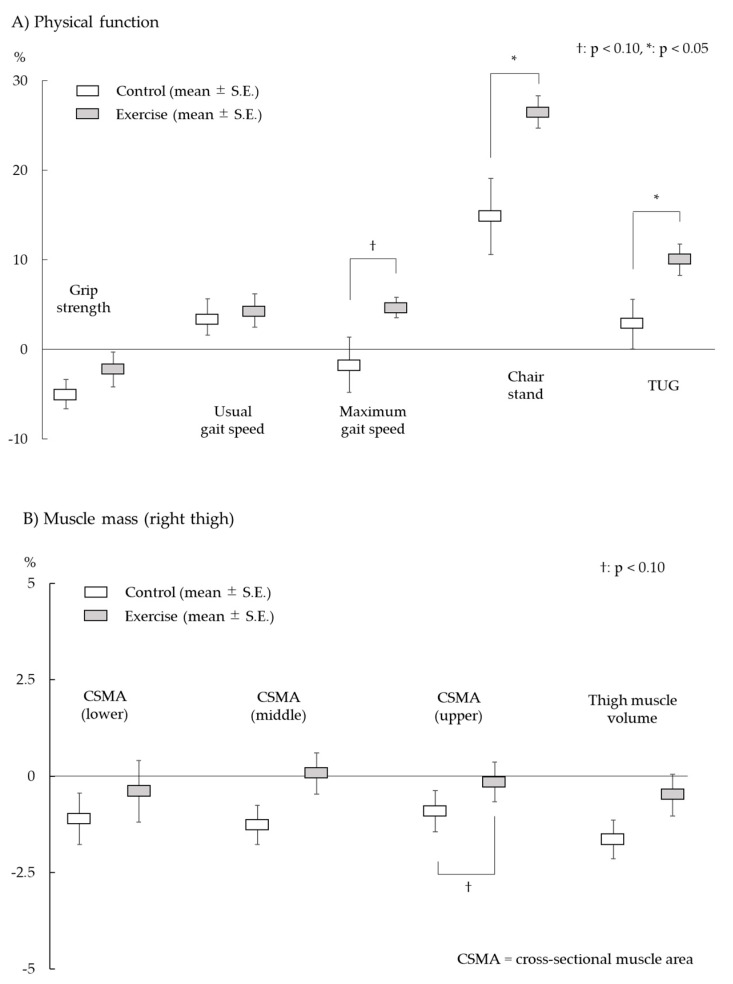
Improvement percentage of sarcopenia-related physical function and muscle mass after intervention.

Table 2 and Figure 3 show the pre- and post-intervention changes in sarcopenia-related physical function in the control and exercise groups. The Student’s *t*-test for paired data in each group showed that grip strength declined significantly post intervention in the control group (*p* = 0.01), but no change was found in the exercise group. There were no significant changes in normal and maximum gait speeds in the control group, while maximum gait speed showed significant improvement in the exercise group post-intervention (*p* < 0.01). The chair stand performance improved in both groups. The exercise group showed significantly better performance on the TUG test post intervention (*p* < 0.01); no changes were seen in the control group. In the repeated-measures ANOVA, significant group-by-time interactions were observed on the chair stand (F = 5.85, *p* = 0.02) and TUG (F = 6.33, *p* = 0.01) tests, with increases in the exercise group. There were no significant group-by-time interactions in the other physical function assessments.

### 3.4. Cross-Sectional Muscle Area/Muscle Volume

Table 3 and Figure 3 show the pre- and post-intervention changes in muscle mass outcomes. There were no significant changes in cross-sectional muscle area and muscle volume in the exercise group. However, the cross-sectional muscle area in the middle (*p* = 0.01) and upper levels (*p* = 0.06) and the muscle volume of the right thigh (*p* < 0.01) declined in the control group post-intervention. Although there was a tendency to prevent loss of muscle mass in the exercise group, no significant interaction effects were detected for cross-sectional muscle area (lower level: F = 0.28, *p* = 0.60; middle level: F = 2.70, *p* = 0.11; upper level: F = 1.05, *p* = 0.31) and muscle volume (F = 1.90, *p* = 0.17). The ASMI also showed no significant group-by-time interaction (F = 1.71, *p* = 0.20).

## 4. Discussion

This RCT indicated that a standardized multicomponent exercise program, including progressive resistance training, improved physical function, especially chair rise and TUG performance, in community-dwelling older adults with sarcopenia or pre-sarcopenia. No adverse events related to the intervention were reported and there was a higher than 80% mean participation rate for the 12 exercise sessions.

Sarcopenia-related physical function and muscle mass decrease with age. Cross-sectional data have indicated an age-associated decline in handgrip strength and muscle mass [28]. In adults aged ≥85 years, as compared with young adults aged 20–29 years, handgrip strength was over 50% lower, and calf muscle cross-sectional area was 15% lower in women and 30% in men [28]. Longitudinal studies also showed that, in individuals aged 75 years, muscle mass decreased at a rate of 0.64%–0.7% in women and 0.8%–0.98% in men per year [29]. Strength was lost more rapidly, at a rate of 3% –4% in men and 2.5%–3% in women per year [29]. Although reduced muscle mass may be an important factor in limited mobility and strength [7], muscle strength as a marker of muscle quality could be more important in estimating mortality risk than is muscle quantity [30]. Therefore, intervention programs are needed to be highly effective at counteracting the decline in physical function, muscle mass and strength associated with ageing. The current RCT indicated useful for improvement of physical function, even for older individuals with sarcopenia. 

Handgrip strength is a good predictor of poor health outcomes, including mortality [30], through mechanisms other than those leading from disease to muscle impairment. Gait speed, chair stand, and TUG tests, is also associated with future adverse outcomes including disability [31,32], hospitalization [33], and mortality [34,35]. A previous intervention study involving eight weeks of high-resistance weight training indicated significant gains in muscle strength and functional mobility among frail residents of nursing homes up to 96 years of age (mean age, 90 ± 1 years) [36]. Thus, it is never too late to start resistance exercise to improve muscle function. Multimodal training is an effective intervention to increase physical capacity among frail older individuals [37]. Integrated care including exercise, nutrition, and psychological interventions improved frailty and sarcopenia status among community-dwelling older adults, with high-intensity training yielding greater improvements [38]. Resistance training, based on the percentage of a maximum of one-repetition maximum showed significant effects on physical variables, whereas resistance training based on the rate of perceived effort presented lower effects [37]. Although a training prescription based on a one-repetition maximum practice could be better for gaining muscle, it is not realistic for determining exercise intensity in the community setting. In the current RCT study, a multicomponent exercise program, including progressive resistance training, mainly used resistance bands, which was not a required prescription based on a one-repetition maximum practice improved physical function in older adults with loss of muscle mass, and could be useful to prevent and improve sarcopenia. However, it did not change muscle volume in older adults with sarcopenia. In order to change muscle volume, stricter exercise protocol may be needed.

A systematic review and meta-analysis that aimed to identify dose-response relationships of resistance training variables to improve muscle strength and morphology in healthy older adults indicated that 50–53 weeks of training is most effective [39]. Our multicomponent exercise program of 12 weekly sessions with a progressive protocol using a resistance band was conducted considering its feasibility in the community. Positive effects on physical function could be expected from our program for older adults with sarcopenia, but it may not be enough (e.g., intensity, frequency, and duration) for increasing muscle mass.

Nutrition may be another key element of multimodal interventions for sarcopenia [39,40]. Malnutrition and dietary patterns contribute to progressive, adverse changes in aging muscle [41,42]. Amino acids, β-hydroxyl β-methyl butyrate, energy, and vitamin D are required for muscle synthesis, so it is possible that nutritional intake influences sarcopenia [43,44]. A review suggested that the benefits of exercise could be enhanced with nutritional supplements (energy, protein, and vitamin D) [44]. On the other hand, previous reviews highlighted the importance of exercise interventions with or without nutritional supplements to improve physical function in community-dwelling older adults with sarcopenia [45]. Our program showed little evidence for increase in muscle mass in those with sarcopenia. A combination of a multicomponent exercise program and nutrition may have positive effects on both physical function and muscle mass among older adults with sarcopenia.

Several factors may mediate the associations of exercise with improvements in muscle strength and mass. For instance, genotypes (e.g., α-actinin-3 gene), endocrine, and lifestyle factors could be associated with age-related decline in muscle function [46,47]. Additional analyses are required to determine the mediation factors that support or limit the effects of exercise on muscle function.

Several limitations of this study should be noted. There was more than 80% of mean participation rate for the 12 exercise sessions. Participants were asked to exercise daily using a booklet and exercise calendar, but their adherence to this was not analyzed. Future studies would greatly benefit from the incorporation of activity monitors on the participants. Although our program mainly focused on resistance training with progressive resistance every two weeks, intensity was determined by perceived exertion, and the process was not standardized. Additionally, although other components, such as aerobic and balance exercises, were included to increase difficulty levels, these progress processes were not constant.

In conclusion, the current RCT suggests that a 12-week multicomponent exercise program with progressive resistance training generally improves physical function in community-dwelling older adults with sarcopenia or pre-sarcopenia. Multicomponent exercise could be effective at counteracting the decline in physical function for sarcopenia. However, it is unclear whether this exercise program is effective in increasing muscle mass among those with sarcopenia. Further studies might be needed to clarify the effect of treatment and prevention for the decline of muscle mass and strength related to aging and sarcopenia.

## Figures and Tables

**Table 1:** Participant characteristics at baseline.

	All (*n* = 72)	Control Group (*n* = 36)	Exercise Group (*n* = 36)	*p*
Age, y, mean ± SD	75.0 ± 6.9	75.8 ± 7.3	74.1 ± 6.6	0.304
Sex, *n* (%)				
Female	51 (70.8%)	25 (69.4%)	26 (72.2%)	0.795
BMI, kg/m^2^, mean ± SD	20.7 ± 2.4	20.6 ± 2.1	20.9 ± 2.7	0.628
Fall history in the past year, *n* (%)	9 (12.5%)	4 (11.1%)	5 (13.9%)	0.722
Medial history, *n* (%)				
Hypertension	25 (35.2%)	10 (27.8%)	15 (42.9%)	0.184
Heart disease	10 (13.9%)	4 (11.1%)	6 (16.7%)	0.496
Diabetes mellitus	6 (8.3%)	4 (11.1%)	2 (5.6%)	0.394
Arthritis	7 (9.7%)	2 (5.6%)	5 (13.9%)	0.233
Medication ^a^, no. mean ± SD	2.6 ± 2.5	2.1 ± 2.2	3.0 ± 2.8	0.285
Sarcopenia status, *n* (%)				
Sarcopenia	20 (27.8%)	11 (30.6%)	9 (25.0%)	0.599
Pre-sarcopenia	52 (72.2%)	25 (69.4%)	27 (75.0%)	
Physical function				
Grip strength, kg, mean ± SD	23.0 ± 5.5	23.2 ± 6.3	22.7 ± 4.6	0.702
Usual gait speed, m/sec, mean ± SD	1.34 ± 0.22	1.35 ± 0.24	1.33 ± 0.18	0.593
Maximum gait speed, m/sec, mean ± SD	1.70 ± 0.28	1.73 ± 0.31	1.67 ± 0.25	0.391
Chair stand ^a^, sec, mean ± SD	10.3 ± 3.2	9.6 ± 2.9	10.9 ± 3.4	0.086
Timed up and go, sec, mean ± SD	8.7 ± 2.0	8.5 ± 2.0	9.0 ± 2.9	0.285
Muscle mass				
ASMI, kg/m^2^, mean ± SD	5.7 ± 0.7	5.7 ± 0.7	5.6 ± 0.8	0.811
Cross-sectional right thigh muscle area ^b^, cm^2^, mean ± SD				
Lower ^c^	47.5 ± 8.2	47.6 ± 8.5	47.3 ± 8.0	0.871
Middle ^c^	64.3 ± 11.0	65.5 ± 11.3	62.8 ± 10.6	0.342
Upper ^c^	79.5 ± 13.9	81.3 ± 14.5	77.4 ± 13.1	0.265
Thigh muscle volume ^b^, cm^3^, mean ± SD	566.1 ± 97.9	574.5 ± 101.9	556.2 ± 93.9	0.455

Data presented as mean ± SD or number (%). There were no significant between-group differences in baseline characteristics. BMI = body mass index; SPPB = short physical performance battery; ASMI = appendicular skeletal muscle mass index. ^a^ Missing, *n* =1. ^b^ Missing, *n* = 7. ^c^ Lower, a 30-slice section from the upper edge of the patella; middle, a 60-slice section from the upper edge of the patella; upper, a 90-slice section from the upper edge of the patella (1 slice = 1.5 mm thickness).

**Table 2:** Changes in sarcopenia-related physical function during the 12-week intervention period.

	Within-Group Differences						Between-Group Differences			
	Control Group (*n* = 36)			Exercise Training Group (*n* = 36)			Control Difference	Intervention Difference	Time by Group Interaction	
	Baseline	At 12 Weeks	*p*	Baseline	At 12 Weeks	*p*			F-Value	*p*
Grip strength, kg	23.2 ± 6.3	22.0 ± 6.3	0.01	22.7 ± 4.6	22.0 ± 4.2	0.09	−1.2 ± 2.2	−0.7 ± 2.4	0.83	0.37
Usual gait speed, m/sec	1.35 ± 0.24	1.39 ± 0.23	0.18	1.33 ± 0.18	1.37 ± 0.14	0.08	0.04 ± 0.15	0.04 ± 0.15	0.10	0.76
Maximum gait speed, m/sec	1.73 ± 0.31	1.75 ± 0.32	0.56	1.67 ± 0.25	1.75 ± 0.24	<0.01	0.02 ± 0.15	0.07 ± 0.12	3.41	0.07
Chair stand ^a^, sec	9.6 ± 2.9	7.6 ± 2.3	<0.01	10.9 ± 3.4	7.9 ± 2.3	<0.01	−1.9 ± 2.0	−3.0 ± 1.7	5.85	0.02
Timed up and go, sec	8.5 ± 2.0	8.2 ± 2.1	0.13	9.0 ± 2.9	8.0 ± 1.5	<0.01	−0.3 ± 1.2	−1.0 ± 1.0	6.33	0.01

Data presented as mean ± SD. ^a^ Missing, *n* = 1 (control group, *n* = 35).

**Table 3:** Changes in muscle mass outcomes during the 12-week intervention period.

	Within-Group Differences					Between-Group Differences				
	Control Group (*n* = 36)			Exercise Training Group (*n* = 36)		Control Difference		Intervention Difference	Time by Group Interaction	
	Baseline	At 12 Weeks	*p*	Baseline	At 12 Weeks	*p*			F-Value	*p*
Cross-sectional right thigh muscle area ^a^, cm^2^										
Lower ^b^	47.6 ± 8.5	47.1 ± 8.5	0.10	47.3 ± 8.0	47.0 ± 7.6	0.49	−0.5 ± 1.9	−0.3 ± 2.1	0.28	0.60
Middle ^b^	65.5 ± 11.3	64.6 ± 11.1	0.01	62.8 ± 10.6	62.8 ± 10.1	0.82	−0.9 ± 1.9	−0.1 ± 2.0	2.70	0.11
Upper ^b^	81.3 ± 14.5	80.5 ± 13.7	0.06	77.4 ± 13.1	77.1 ± 12.6	0.53	−0.8 ± 2.6	−0.3 ± 2.3	1.05	0.31
Thigh muscle volume ^a^, cm^3^	574.5 ± 101.9	565.0 ± 100.5	<0.01	556.2 ± 93.9	552.6 ± 89.3	0.29	−9.5 ± 17.2	−3.5 ± 17.7	1.90	0.17

Data presented as mean ± SD. ^a^ Missing, *n* =7 (control group, *n* = 35; exercise training group, *n* = 30). ^b^ Lower, a 30-slice section from the upper edge of the patella; middle, a 60-slice section from the upper edge of the patella; upper, a 90-slice section from the upper edge of the patella (1 slice = 1.5 mm thickness).
